# Characterization of the distribution and dynamics of chromatin states in the *C. elegans* germline reveals substantial H3K4me3 remodeling during oogenesis

**DOI:** 10.1101/gr.278247.123

**Published:** 2024-01

**Authors:** Mariateresa Mazzetto, Lauren E. Gonzalez, Nancy Sanchez, Valerie Reinke

**Affiliations:** Department of Genetics, Yale University, New Haven, Connecticut 06520, USA

## Abstract

Chromatin organization in the *C. elegans* germline is tightly regulated and critical for germ cell differentiation. Although certain germline epigenetic regulatory mechanisms have been identified, how they influence chromatin structure and ultimately gene expression remains unclear, in part because most genomic studies have focused on data collected from intact worms comprising both somatic and germline tissues. We therefore analyzed histone modification and chromatin accessibility data from isolated germ nuclei representing undifferentiated proliferating and meiosis I populations to define chromatin states. We correlated these states with overall transcript abundance, spatiotemporal expression patterns, and the function of small RNA pathways. Because the essential role of the germline is to transmit genetic information and establish gene expression in the early embryo, we compared epigenetic and transcriptomic profiles from undifferentiated germ cells to those of embryos to define the epigenetic changes during this developmental transition. The active histone modification H3K4me3 shows particularly dynamic remodeling as germ cells differentiate into oocytes, which suggests a mechanism for establishing early transcription of essential genes during zygotic genome activation. This analysis highlights the dynamism of the chromatin landscape across developmental transitions and provides a resource for future investigation into epigenetic regulatory mechanisms in germ cells.

Epigenetic mechanisms are essential to correctly establish tissue-specific gene expression programs during development. At the genomic level, such mechanisms include the deposition of histone modifications and histone variants that lead to alterations in chromatin structure and organization, which can have both global and local effects on gene expression. Small RNA pathways also mediate epigenetic information by influencing transcript stability and protein translation, as well as feeding back to the nucleus to affect chromatin states at target loci.

Epigenetic mechanisms are especially critical in germ cells, which have the task of transmitting both genetic and epigenetic information from parent to progeny. In *Caenorhabditis elegans*, multiple epigenetic mechanisms are implicated in different aspects of germ cell differentiation and function. These mechanisms include the mutually exclusive relationship of the repressive H3K27me3 and active H3K36me3 histone modifications, which reinforce permissive chromatin environments on autosomes (Gaydos et al. 2012; Meers et al. 2017; Li et al. 2019), and enrich repressive marks on the X Chromosome, leading to its inactivation (Bender et al. 2004; van Mierlo et al. 2019). The relationship of these two histone modifications with other modifications in the germline is poorly understood. Additionally, several distinct small RNA pathways are very active in germ cells and affect the stability of target transcripts as well as select histone modifications at target loci (Frolows and Ashe 2021; Seroussi et al. 2023). However, the extent to which the target gene chromatin state is altered as a consequence of the activity of individual small RNA pathways has not been systematically determined. Finally, although epigenetic information is considered critical for highly differentiated gametes to reactivate totipotency in the zygote and initiate successful embryogenesis in the absence of active transcription, the accompanying changes to chromatin structure and how those changes contribute to correct genome activation in the early embryo are not well characterized (Robert et al. 2015).

Functional and genomic studies have revealed some epigenetic regulatory mechanisms that influence germline chromatin organization and establish reproducible gene expression programs. However, the field has been limited by the fact that most genomic studies in *C. elegans* have been performed on whole animals, which simultaneously capture both germline and somatic tissues and therefore can obscure germline-specific mechanisms. We recently addressed this limitation by developing a protocol to isolate undifferentiated germ nuclei (IGN) at sufficient numbers that allow for genomic assays (Han et al. 2019). Over the past few years, we and others have successfully used this protocol for different high-throughput sequencing assays in IGN, such as transcriptome profiling (RNA-seq), histone modification profiling via chromatin immunoprecipitation (ChIP-seq), and chromatin accessibility profiling via assay for transposon-accessible chromatin (ATAC-seq) (Sanchez et al. 2023).

In this study, we characterized *C. elegans* chromatin regulatory mechanisms in IGN using these data sets to define diverse chromatin states in the germline genome, reveal intra- and inter-chromosomal patterns of histone modifications and chromatin accessibility, and identify chromatin states that are unique to the germline relative to the soma. Germline-specific chromatin state data permit investigation of the environment surrounding genes expressed throughout germline development, as well as the role of small RNA regulation in establishing the chromatin environment of target loci. Moreover, the identification of histone modifications that show particularly dynamic remodeling as germ cells differentiate into oocytes determines the gene targets of those modification(s) as well as the effect on gene expression in the very early embryo. Overall, such analyses improve our understanding of the dynamic chromatin state found in germ cells and provide a platform for continued investigation into global and local epigenetic germline regulatory mechanisms.

## Results

### Characterization of germline chromatin states

To characterize the distribution of regulatory states of *C. elegans* germline chromatin in wild-type adult hermaphrodites, we analyzed epigenetic data from IGN, which primarily comprise undifferentiated proliferating and early meiotic germ cells (Supplemental Fig. S1A; Han et al. 2019). Data sets include profiles of five well-characterized histone marks (H3K27me3, H3K9me3, H3K36me3, H3K4me3, and H3K27ac), transcript abundance, and chromatin accessibility data (Methods). To identify active or inactive genome regions that correlate with particular combinations of histone modifications and associate these data with genomic features such as promoters, enhancers, exons, and introns, we annotated chromatin states across the genome using ChromHMM (Ernst and Kellis 2012). This program discovers chromatin state signatures using a hidden Markov model (HMM) that measures the presence or absence of each mark, singly or in combination, across genomic features to define functionally distinct chromatin states. ChromHMM also provides the quantified distribution (“coverage”) of each state across the genome.

**Figure 1.**
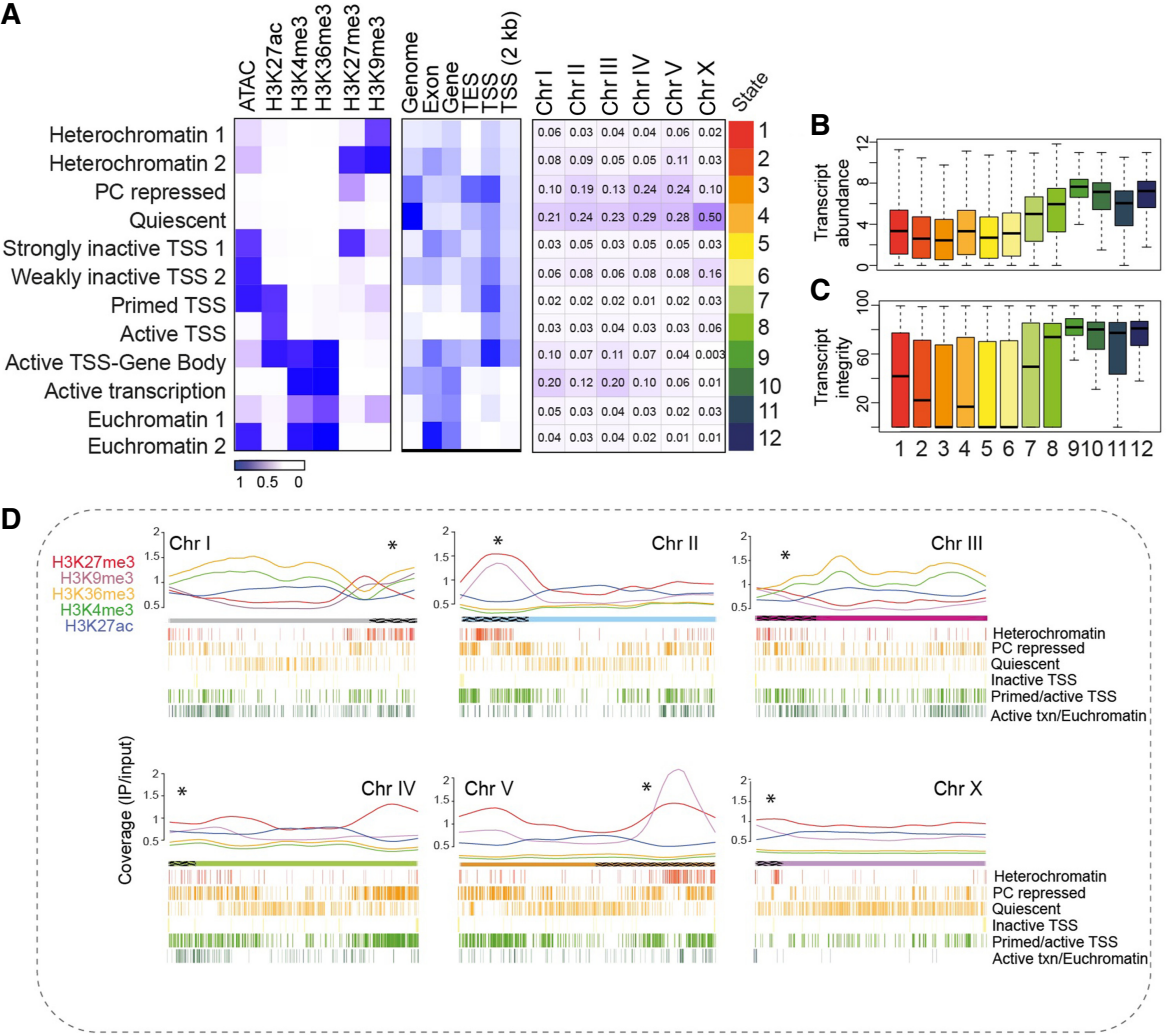
Characteristics of germline chromatin states. (*A*) Annotated chromatin states from isolated germ nuclei (IGN). (*Left*) The probability of each histone mark or open chromatin for each of the 12 defined states (as a range from zero to one) is plotted as a heatmap. (*Middle*) The genomic coverage across gene features for each state. (*Right*) The percentage of each chromosome in each state. (*B*) Transcript abundance (as log FPKM) of the genes located within each chromatin state. (*C*) Percentage of transcript integrity, which measures how prone to degradation a transcript is, of the genes within each chromatin state. (*D*) Distribution of individual histone modifications along each chromosome, plotted as the normalized signal (IP/input ratio). Asterisks *above* each chromosome indicate the end at which the pairing center is located, and the hatching on the chromosome indicates the extent of the pairing center. The 12 chromatin states were condensed into six groups of functionally related marks, with the distribution across each chromosome represented by hash marks *underneath*.

We defined 12 chromatin states characterized by specific combinations of histone marks and distinct distributions relative to genomic features (Fig. 1A; Supplemental Fig. S1B,C; Supplemental File S1). Six of the states represent repressed or inactive chromatin. We named the first state “heterochromatin 1,” because it had high levels of H3K9me3. The second state, “heterochromatin 2” is characterized by high levels of both H3K9me3 and H3K27me3. The third state had high levels of H3K27me3 alone and is called “Polycomb (PC) repressed,” whereas state 4 had low levels of any mark and is termed “quiescent.” States 5 and 6 combine high chromatin accessibility and the presence of either high or low H3K27me3 signal around the transcription start site (TSS) and are labeled “strongly inactive TSS” and “weakly inactive TSS,” respectively (Fig. 1A). For the six states representing active chromatin, state 7 shows accessible chromatin and both active and repressive marks distributed around the TSS and is therefore termed “primed TSS.” State 8, active TSS, is represented primarily by high levels of H3K27ac near the TSS, whereas state 9, active TSS–gene body features high H3K27ac at the TSS as well as H3K4me3 and H3K36me3 across the gene body. Genomic regions in state 10 show high levels of H3K36me3 and H3K4me3 on gene bodies without high H3K27ac and are named “active transcription.” Finally, states 11 and 12 have both H3K4me3 and H3K36me3 and either low or highly accessible chromatin and are called “euchromatin 1 and 2,” respectively.

Relative to genes in inactive or repressive chromatin states, genes associated with active chromatin states (states 7–12) were characterized by overall higher transcript abundance (Fig. 1B) and transcript integrity, which measures how prone a transcript is to degradation (Fig. 1C; Wang et al. 2016). In addition, Gene Ontology (GO) analysis for the genes associated with each chromatin state was enriched for developmental and germline-related categories in active states and neuronal-related categories in inactive states; glycoprotein metabolism was uniquely enriched in state 2, heterochromatin 2 (Supplemental Fig. S1C). These observations show that germline chromatin can be segmented into regulatory states with distinct features that reflect known gene function and gene expression in that tissue.

In the germline, autosomes are generally well expressed and enriched with active marks, whereas the X Chromosome is largely silenced, with enriched H3K27me3 and low levels of active marks (Bender et al. 2004, 2006; Gaydos et al. 2012; Meers et al. 2017; Li et al. 2019; van Mierlo et al. 2019). Additionally, meiotic recombination events are concentrated toward the ends of autosomes but are broadly distributed along the length of the X Chromosome (Barnes et al. 1995; Liu et al. 2011). We compared IGN chromatin state and histone modification patterns to identify both inter- and intra-chromosomal differences at higher resolution. The coverage of each chromatin state relative to the percentage of chromosome length (Fig. 1A, right panel) shows that the autosomes display functionally diverse (inactive, quiescent, and high transcription) states. In contrast, the X Chromosome displays a quiescent or inactive state for 84% of its length, a scattered distribution of heterochromatin states, low levels of active marks, and higher levels of the H3K27me3 mark compared with active marks (Fig. 1D).

We also observed distinct intra-chromosomal patterns of chromatin states and histone modification distributions (Fig. 1D). Although heterochromatin is preferentially concentrated on chromosomal arms, euchromatin is mainly distributed on the central part of each chromosome, which are generally gene dense and enriched for essential genes (The *C. elegans* Sequencing Consortium 1998). In addition, heterochromatin states and repressive histone marks, especially H3K27me3, tend to be distributed around the meiotic pairing center, whereas active marks are depleted, as previously reported (Liu et al. 2011). In addition to these general, expected patterns, each chromosome has unique aspects of chromatin state distribution. For example, Chromosome III has relatively low levels of repressive marks across the whole chromosome length, even on the chromosomal arms. Additionally, Chromosome IV does not show specific changes in levels of histone marks at the pairing center, perhaps owing to having relatively few pairing center sequence motifs (Phillips et al. 2009; Liu et al. 2011). Chromosome IV also displays high levels of H3K27me3 at the right arm of the chromosome, which is the site of a 3-Mb domain containing thousands of tiny genes encoding type I piRNAs. Enrichment of H3K27me3 on genes encoding this class of small RNAs was previously observed in whole-animal ChIP-seq analysis (Beltran et al. 2019). Finally, a notably strong H3K9me3 signal on Chromosome V correlates with a genomic region enriched for pseudogenes and G protein–coupled receptor genes with neuron-restricted expression.

With these observations, we show that annotation of chromatin states using purified germ nuclei gives a comprehensive characterization of chromatin regulatory states among and within chromosomes in the *C. elegans* germline.

### Characterization of somatic chromatin states relative to the germline

**Figure 2.**
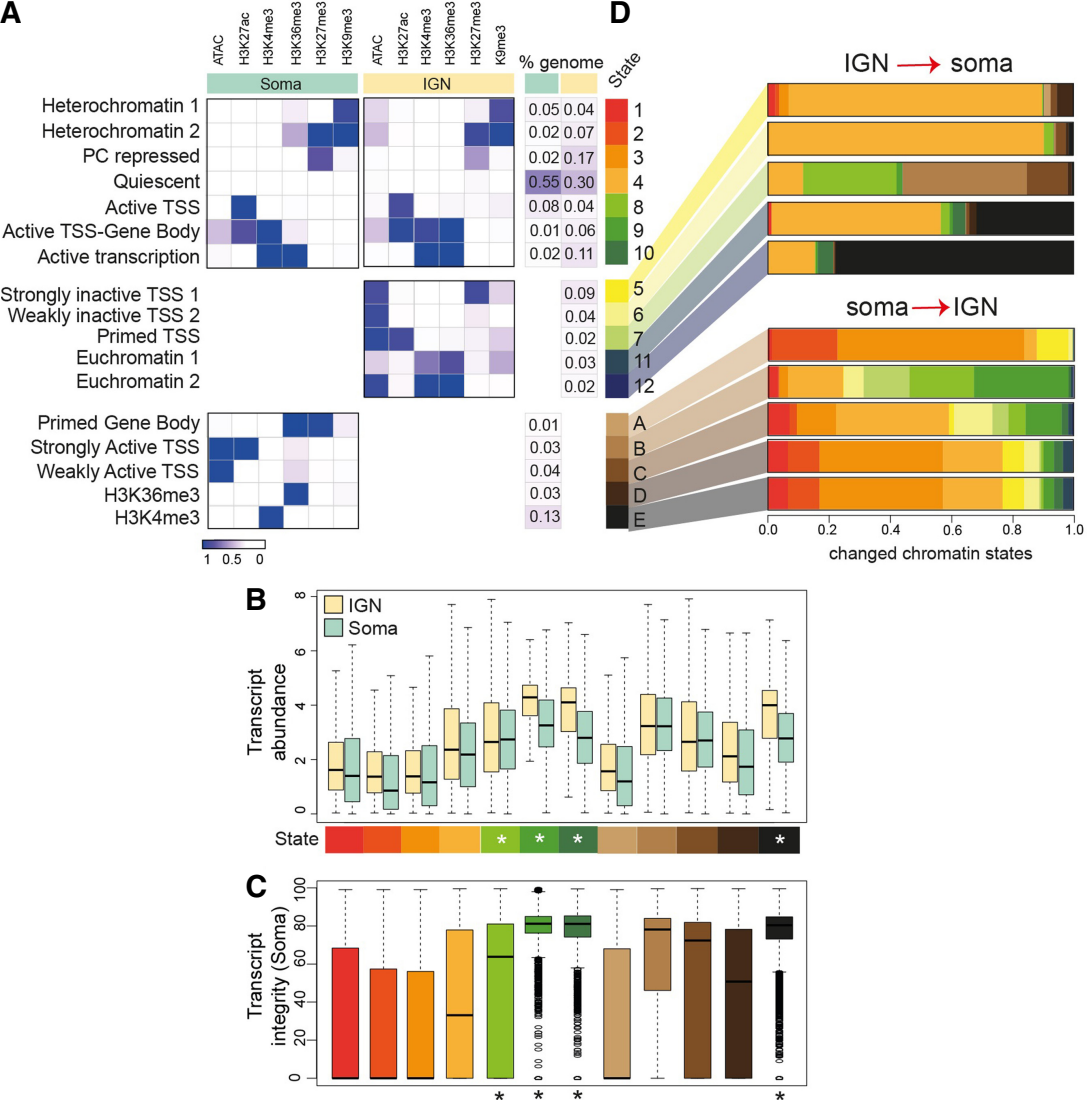
Chromatin states unique to IGN and soma. (*A*) Annotated chromatin states from adult mixed somatic cells compared with germline states from Figure 1, divided into “shared” states (*above*), “only-IGN” states (*center*), and “only-soma” states (labeled A–E in shades of brown; *below*). The probability of each histone mark or open chromatin for each of the 12 defined states (as a range from zero to one) is plotted as a heatmap, with the percentage of each chromosome in each state at *left*. (*B*) Transcript abundance (as log FPKM) of the genes located in each chromatin state found in soma. Asterisks mark active states. (*C*) Percentage of transcript integrity of the genes located in each chromatin state found in the soma. (*D*) Altered chromatin state distribution between IGN and soma. For the cumulative fraction of the genome with each unique chromatin state in IGN (5, 6, 7, 11, and 12) or soma (A–E), the shifts to different chromatin states in the opposing tissue are displayed as a barplot, where each color corresponds to a previously defined chromatin state (e.g., IGN-specific state 12 [2% of the genome] mostly [80%] adopts soma-specific state E in the soma).

To define unique aspects of the germline chromatin landscape, we wished to contrast chromatin states with those in somatic tissues. We therefore computed chromatin states for adult *glp-1*-mutant worms, which lack a germline and represent only somatic tissues, using published epigenetic and transcriptomic data representing the same chromatin features analyzed in the germline (Jänes et al. 2018; Han et al. 2019). We again classified 12 chromatin states characterized by specific combinations of histone marks and distinct distributions along the genome (Fig. 2A; Supplemental Fig. S2A). Similar to the germline, genes associated with active chromatin states in the soma were characterized by higher transcript abundance (Fig. 2B) and transcript integrity (Fig. 2C), whereas genes contained in quiescent or inactive chromatin states were less abundant and more prone to degradation. Unlike the germline, the X Chromosome in the soma is not enriched for inactive states (Fig. 2A; Supplemental Fig. S2A). Moreover, the levels of active marks are generally lower along all chromosomes in the soma, whereas H3K27me3 is particularly high over the length of each chromosome, with the exceptions of Chromosomes I and III (Supplemental Fig. S2B). High levels of H3K27me3 might be the result of mixing multiple cell types, in which tissue-specific genes would be inactive in most tissues, leading to a higher H3K27me3 signal on average along the chromosome. GO analysis revealed an enrichment in active states for developmental and proliferation categories, gene categories that likely span multiple tissue types (Supplemental Fig. S2C). Heterochromatin regions instead showed an enrichment for chemosensory pathways and immune response categories, which are likely primarily involved in specific cell types and therefore need to be repressed in other cell types. Overall, these chromatin states from whole somatic tissue provide a useful platform to contrast with germline chromatin states.

We therefore directly compared chromatin states between IGN and soma to identify germline-specific aspects of chromatin regulation. Seven somatic chromatin states are very similar to states 1–4 and 8–10 in IGN, which we consider “shared” states: two heterochromatin states, as well as the PC repressed, quiescent, active TSS, active TSS–gene body, and active transcription states, which together represent 75%–80% of the genome in each soma and IGN (Fig. 2A). For the remaining 20%–25% of the genome, both IGN and soma show unique chromatin states. For example, in IGN, ∼9% of the genome shows the silencing mark H3K27me3 at promoters in sites of open chromatin (strongly inactive TSS state 5), a state that is not found in the soma. In contrast, 16% of the genome in the soma have a single active histone mark (H3K36me3 or H3K4me3) (Fig. 2A, states D and E), whereas those two marks are always found together in IGN chromatin states.

We then investigated how the distribution of these chromatin states changed between IGN and soma at specific regions of the genome; even for a state present in both germline and soma, the specific regions of the genome marked by that state could differ between the two tissues. Overall, ∼42% of the genome changed chromatin state between IGN and soma, with some interesting specific alterations between states (Fig. 2D). For example, regions marked by the germline-specific “strongly inactive TSS” state 5 are found largely in the quiescent state in the soma. Regions marked by the “H3K36me3 only” state D in the soma generally lose H3K36me3 and become quiescent (state 4) or PC repressed (state 3) in IGN, whereas regions in the “H3K4me3-only” state E gain H3K36me3 and become the active states 9 (TSS–gene body) and 10 (active transcription) in IGN. At the resolution of individual genes, we also found many changes between germline and somatic epigenetic profiles (Supplemental Fig. S2D–F). For instance, *bath-4*, a germline-specific gene enriched in germline precursor cells, shows strong enrichment for active marks in the germline but enrichment for H3K27me3 in the soma. Notably, H3K36me3 and H3K27me3 are mutually exclusive in IGN but show some overlap at individual genes in the soma (Supplemental Fig. S2G), which could again be related to mixing multiple cell types. In sum, comparing IGN chromatin states with those found in the soma reveals both shared and distinct epigenetic profiles, suggesting that there are unique aspects to epigenetic regulation and/or function in the germline.

### Using chromatin states to understand germline regulatory mechanisms

The *C. elegans* germline is characterized by an organized trajectory along which proliferating germ cells enter meiosis I and either differentiate into sperm at the L4 stage or into oocytes as adults. These temporal and spatial dynamics result in extensive differential gene regulation (Seydoux and Schedl 2001; Reinke et al. 2004; Kimble and Crittenden 2007) that defines groups of genes with enriched expression before differentiation (“pregametic”), during spermatogenesis, or during oogenesis (Lee et al. 2012). To better understand the relationship between chromatin states, gene regulation, and germline differentiation, we examined the IGN chromatin state distribution specifically associated with these gene sets.

**Figure 3.**
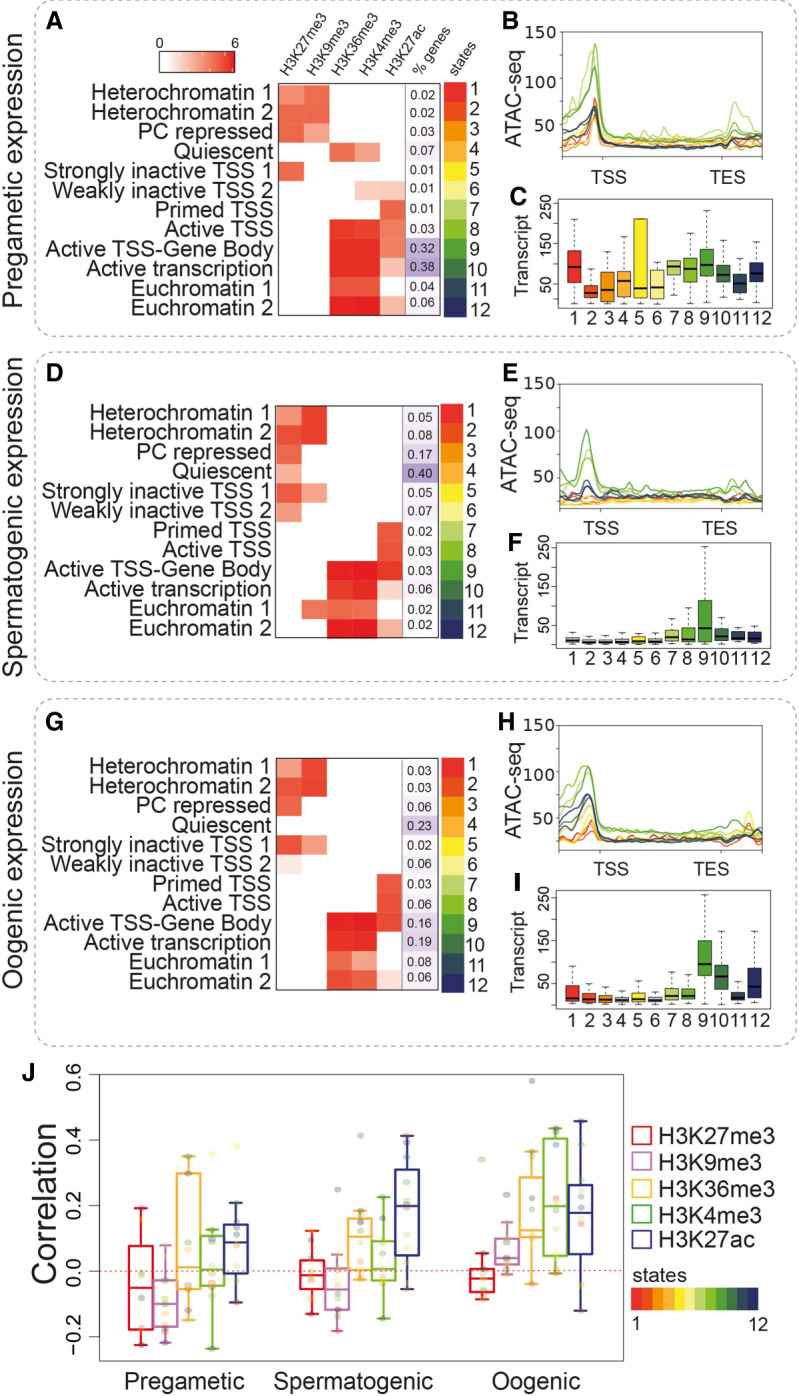
Chromatin states of classes of germline-expressed genes in IGN. (*A*–*C*) Pregamete-enriched gene class, (*D*–*F*) spermatogenesis-enriched gene class, and (*G*–*I*) oogenesis-enriched gene class (Lee et al. 2017). (*A*,*D*,*G*) Heatmap of histone mark coverage in each state, with the percentage of genes in each state to the *right*. (*B*,*E*,*H*) Chromatin accessibility across chromatin states. Each line represents a chromatin state by assigned color. Normalized signals (as IP/input ratio) were plotted across the upstream region (TSS − 2000 bp), gene body, and downstream region (TES + 2000 bp). (*C*,*F*,*I*) Transcript abundance as FPKM. Values related to each state are plotted. (*J*) Pearson's correlation between transcript abundance (FPKM) in IGN and histone mark levels (as normalized IP/input signal) for spermatogenic, oogenic, and pregametic gene classes. Each point represents a single chromatin state with the color code as in Figure 1.

Of the three groups of genes, the pregametic genes are best represented by IGN, which primarily captures nuclei from morphologically undifferentiated germ cells in the distal proliferative and medial pachytene regions of the adult germline (Han et al. 2019). We found that 70% of pregametic genes are in states 9 and 10, active TSS–gene body and active transcription, with high levels of H3K36me3, H3K4me3, and H3K27ac (Fig. 3A; Supplemental Fig. S3A) and high transcript abundance (Fig. 3C). Surprisingly, genes in these states do not have the most open chromatin; instead, states 7 and 8, primed TSS and active TSS, which represent only 4% of the pregametic genes, have substantially more open chromatin (Fig. 3B). This result suggests that many genes can be abundantly expressed even if the chromatin is not maximally accessible. For instance, 16% of pregametic genes are associated with inactive states 1–6, yet show at least some level of expression, with particularly high transcript abundance for state 1 (heterochromatin 1), which is characterized by both H3K9me3 and H3K27me3 but has open chromatin at the TSS that is equivalent to that of euchromatin states 11 and 12 (Fig. 3B,C). High transcript abundance could be owing to post-transcriptional stabilization of mRNAs produced during larval germline development rather than ongoing transcription; however, the open chromatin at the promoter suggests the possibility of transcription from a heterochromatic environment. Of the 28 genes in this state, most have unknown or uncharacterized function, but a few are known or are expected to act during germ cell development, such as *zim-1*, *hcp-2*, *eif-3.K*, and *eri-5* (Supplemental Fig. S3B–D).

We then focused on how the spermatogenesis gene class is regulated in adult IGN. These genes are expected to be inactive because spermatogenesis stops abruptly at the end of L4 upon the switch to oogenesis in hermaphrodites (Reinke et al. 2004). As expected, 82% of spermatogenic genes are found in inactive chromatin states 1–6, all of which have moderate to high levels of H3K27me3, suggesting that this mark is a key component involved in silencing these genes (Fig. 3D; Supplemental Fig. S4A). In addition, genes involved in spermatogenesis are mostly in a closed chromatin configuration (Fig. 3E) with low transcript abundance (Fig. 3F). Recently, the piRNA pathway (Ashe et al. 2012; Bagijn et al. 2012; Wang and Lin 2021) was shown to down-regulate spermatogenesis-expressed genes in the adult germline, dependent on the nuclear Argonaute (AGO) protein HRDE-1 (Cornes et al. 2022). HRDE-1 is expected to recruit H3K9me3 to target genes, although this outcome was not directly tested. Only 13% of all spermatogenesis genes are associated with H3K9me3 (states 1 and 2), but 30% of piRNA target genes are in states 1 and 2 and are marked by H3K9me3 (Supplemental Fig. S4B). The piRNA pathway also targets pseudogenes, and 12 of 23 pseudogenes with spermatogenesis-enriched expression are in states 1 and 2, a fourfold enrichment. Together, these results support the idea that at least a subset of piRNA target genes expressed during spermatogenesis are likely to be silenced through recruitment of H3K9me3.

Unexpectedly, spermatogenesis genes in state 9, active TSS–gene body, show particularly high transcript abundance and open chromatin at the TSS, suggesting that they are still expressed or stabilized in adult germ cells. We found that genes in this and similar states do not show a spike of expression at the L4 stage, as seen for other spermatogenesis genes, but instead have consistent expression across development (Supplemental Fig. S4C), as well as similar levels between hermaphrodite soma and males at the L4 stage (Supplemental Fig. S4D), indicating that their expression is not restricted to spermatogenesis but occurs in multiple tissues. Consistent with this possibility, GO analysis revealed that these genes are enriched for functions in basic metabolism (Supplemental Fig. S4E) and thus are likely to be expressed at other times and places, as well as during spermatogenesis.

Genes in the oogenesis gene class are distributed more evenly among chromatin states than either the pregametic or spermatogenesis groups, with 43% in inactive states (1–6) and 57% in active states (7–12) in adult IGN (Fig. 2G; Supplemental Fig. S5A). We speculated that oogenesis genes in inactive chromatin states might not become expressed until oocytes start to form, whereas genes in active states are expressed in IGN. To explore this possibility, we examined oogenesis genes located on the X Chromosome, which are specifically up-regulated in developing oocytes in the proximal germline (Kelly et al. 2002; Tabuchi et al. 2018). A total of 60% of X-linked oogenesis genes are in the inactive states 1–6, with 34% found in state 4, quiescent, which has very low levels of any modification (Supplemental Fig. S5B). Indeed, almost half (47%) of oogenesis genes in the quiescent state are located on the X. However, 40% of X-linked oogenesis genes are in active states 7–12. Thus, even though the expression of most X-linked oogenesis-enriched genes is restricted to the proximal germline, they can show active, quiescent, or inactive states in IGN. Specific examples highlight the heterogeneity found in oogenesis genes (Supplemental Fig. S5C–E). For genes found in states 8 (active TSS), 9 (active TSS–gene body), 10 (high transcription), and 12 (euchromatin), GO analysis revealed an enrichment for categories related to gamete generation and reproduction (Supplemental Fig. S5F); however, overall, a wide variety of gene functions are found in the different chromatin states. Together, these observations point to diverse regulatory mechanisms beyond the chromatin state that combine to determine expression levels of genes in the oogenesis-enriched class.

We also noted common trends for all three groups of genes with germline-enriched expression. Chromatin state 7, primed TSS, which represents only 1%–3% of genes in each group and is specifically marked by H3K27ac but no other active modifications, consistently has the most open chromatin conformation (Fig. 3B,E,H). However, open chromatin does not directly correlate with transcript levels, as genes in this state typically have lower transcript abundance compared with that of other active states with less open chromatin (Fig. 3C,F,I). Instead, peak transcript abundance consistently occurs in state 9, active TSS–gene body, which is characterized by high levels of all three activating marks H3K36me3, H3K4me3, and H3K27ac with only an intermediate level of open chromatin. Overall, transcript abundance in all three categories is most consistently correlated with H3K27ac, whereas the expression of oogenesis-expressed genes in IGN is additionally positively correlated with H3K36me3 and H3K4me3 levels (Fig. 3J). In sum, diverse chromatin environments in IGN at genes with spatial and temporal regulation during germ cell differentiation reveal complex relationships between chromatin state and transcript abundance and suggest the possibility of transcription from heterochromatic regions, as well as a role for post-transcriptional regulation.

### Chromatin state of gene targets of small RNA pathways

The disconnect between chromatin state and transcript abundance for many of the pregametic, spermatogenesis, and oogenesis-expressed genes suggests a contribution from post-transcriptional regulatory mechanisms. In *C. elegans*, diverse small RNA pathways are key post-transcriptional mediators of gene regulation, especially in the germline. These germline small RNA pathways typically affect transcript stability, but some have also been implicated in altering chromatin state at target genes as a downstream effect (Frolows and Ashe 2021). However, whether particular chromatin states characterize specific pathway target genes has not been systematically investigated specifically in germ cells. Classes of small RNAs are primarily defined by their associated AGO protein, which dictates their subcellular localization and accessibility to targets (Dueck and Meister 2014). A recent study defined four distinct groups of AGO proteins based on shared targets and overlapping functions and phenotypes: the germline-specific CSR-1 and WAGO-1 groups, the sperm-enriched ALG-3/4 group, and the somatic ERGO-1 group (Seroussi et al. 2023).

To investigate the relationship between epigenetic state and these functionally distinct AGO/small RNA pathways, we first examined the distribution of germline chromatin states around the gene targets of each AGO in each subgroup. We found that ∼80% of the targets of each AGO in the CSR-1 group (CSR-1, VRSA-1, and WAGO-4) are associated with active chromatin states, as expected given that the CSR-1 pathway primarily targets germline-expressed genes (Supplemental Fig. S6A). However, AGOs in the ALG-3/4 group (ALG-3, ALG-4, RDE-1, and WAGO-10) have a more variable distribution, with ALG-3/4 target genes distributed roughly evenly between active and inactive chromatin states, whereas ∼80% of RDE-1 and WAGO-10 target genes are associated with inactive chromatin states (Supplemental Fig. S6A). Targets of the ERGO-1 group, which primarily represents somatic genes not expressed in the germline (Seroussi et al. 2023), are mostly associated with inactive states. Similarly, AGOs in the WAGO-1 group are important for the repression of transposable elements (Seroussi et al. 2023), and the gene targets are mostly in inactive chromatin states. Target genes of the CSR-1 and ALG-3/4 groups have higher transcript abundance in the IGN compared with the soma, whereas the targets of the ERGO-1 and WAGO-1 groups show similar levels of transcript abundance in the IGN and soma (Supplemental Fig. S6B).

**Figure 4.**
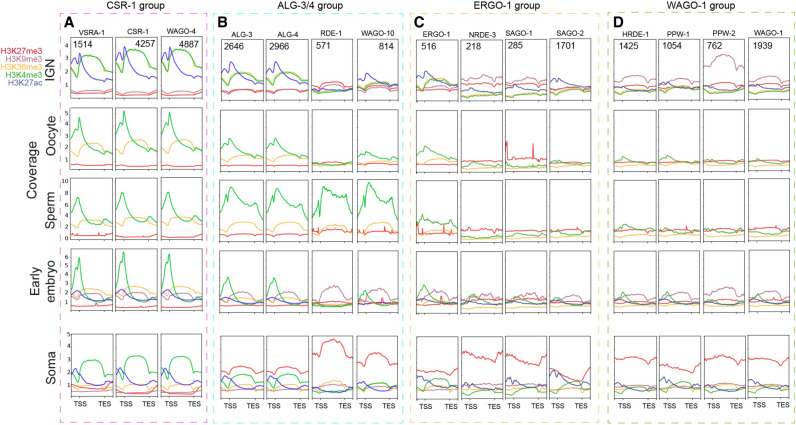
Chromatin states of AGO target genes. (*A*–*D*) Metagene plots of the individual histone marks around target genes of the CSR-1 (*A*), ALG-3/4 (*B*), ERGO-1 (*C*), and WAGO-1 (*D*) groups for IGN, oocyte, sperm, early embryo, and soma data sets. Normalized signals (as IP/input ratio) were plotted for the upstream region (TSS − 1000 bp), gene body, and downstream region (TES + 1000 bp).

To further explore the specific histone marks associated with different sets of AGO targets and to follow their dynamics during gametogenesis and the onset of embryogenesis, we compared data sets from IGN, oocytes, sperm, early embryos, and soma (Methods). For each timepoint, at least three histone modifications were assessed (Fig. 4A–D). Target genes of the CSR-1 group have similarly high levels of H3K4me3 and H3K36me3 in IGN, but H3K36me3 levels specifically drop in gametes and remain low in embryos and soma, whereas H3K4me3 remains high on these genes (Fig. 4A). This observation suggests that the CSR-1 small RNA pathway plays a role in reinforcing the H3K36me3 pattern at these genes specifically in the germline. In comparison, targets of the ALG-3/4 group have low levels of active marks in IGN but a strong enrichment of H3K4me3 specifically in sperm (Fig. 4B), indicating that ALG-3/4 might promote deposition of this mark during spermatogenesis (Seroussi et al. 2023). These target genes also have elevated H3K27me3 in the soma, especially the targets of RDE-1 and WAGO-10, suggesting they are specifically targeted for silencing during or after embryogenesis. In contrast, target genes of the ERGO-1 and WAGO-1 groups tend to have relatively low levels of all marks in IGN and gametes, with the exception of H3K9me3, which is particularly high for PPW-1 (Fig. 4C,D). H3K9me3 is detectable in early embryos for targets of all AGOs in the WAGO-1 subgroup, suggesting it persists through gametogenesis (H3K9me3 profiles are not available for oocytes or sperm). However, in soma, H3K27me3 becomes the dominant silencing mark for these targets, suggesting a major shift during development in the mode of gene silencing at targets of the WAGO-1 group. In sum, gene targets of different small RNA pathways show specific and dynamic epigenetic profiles in the germline and during development, indicating that post-transcriptional regulation of mRNAs by these pathways is broadly reflected at the level of chromatin.

### H3K4me3 is dynamically redistributed in the maternal-to-embryo transition

**Figure 5.**
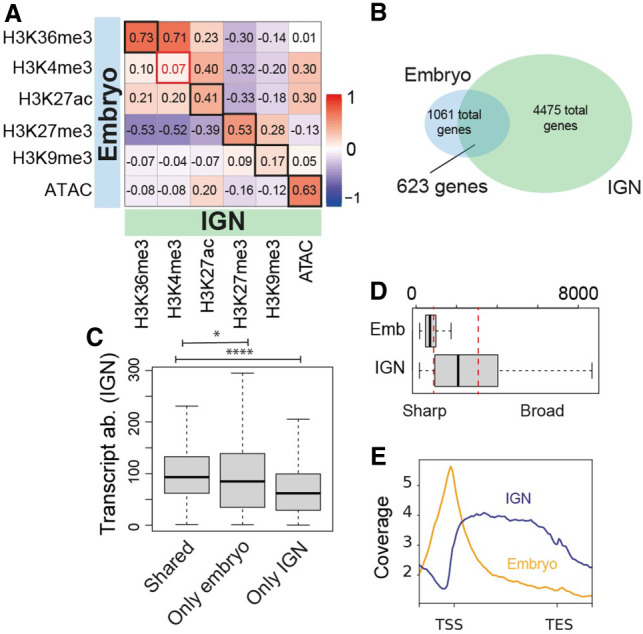
H3K4me3 distribution differs between IGN and embryo. (*A*) Pearson's correlation plot for IGN (horizontal) versus embryo (vertical) for each histone modification. (*B*) Overlap of genes marked by H3K4me3 in IGN versus embryos. Significance assessed by hypergeometric distribution. (*C*) Transcript abundance in IGN for IGN-specific, embryo-specific, and shared genes. *P*-value legend: (*) <0.05, (**) <0.005, (***) <0.0005, (****) <0.0005. (*D*) Breadth of H3K4me3 peaks in embryos (*left*) and IGN (*right*). (*E*) Metagene plots of H3K4me3 signal (as normalized IP/input ratio) around genes marked by H3K4me3 in IGN and embryo.

The dynamic chromatin profiles in IGN, gametes, and embryos at target genes of small RNA pathways prompted us to further investigate chromatin remodeling genome-wide across the maternal-to-embryo developmental transition. We initially compared the genome-wide distribution of histone modifications in IGN to a published mixed-stage embryo data set (Methods). Individual histone modifications correlated well between IGN and mixed-stage embryos (r > 0.20), with the striking exception of H3K4me3 (r = 0.07) (Fig. 5A). The low correlation is partly a result of H3K4me3 marking different genes in the two data sets (Fig. 5B). IGN have many more genes with significant levels of H3K4me3 than do embryos, indicating extensive remodeling during the germline-to-embryo transition. Conversely, more than half of the genes associated with H3K4me3 in the embryo were also associated with this mark in IGN, suggesting that maternal H3K4me3 contributes significantly to the embryonic H3K4me3 pattern. Genes with H3K4me3 in both IGN and embryos have a higher transcript abundance than those marked at only one stage (Fig. 5C). GO analysis showed that genes marked by “H3K4me3 only” in the IGN or embryos reflect germline or embryogenesis functions, respectively, whereas shared genes represent housekeeping and metabolic genes (Supplemental Fig. S7A).

H3K4me3 is most often found at a relatively narrow peak located at promoters but has recently been found in a broad pattern in mouse oocytes and during aging in the *C. elegans* soma (Dahl et al. 2016; Pu et al. 2018). We therefore measured H3K4me3 peak breadth and found that H3K4me3 peaks were significantly broader in IGN than in embryos (Fig. 5D). Broad peaks (>3000 bp) were only observed in IGN and represent 35% of total IGN H3K4me3 peaks. Conversely, sharp peaks (<1000 bp) represent 77% of embryonic peaks but only 27% of IGN peaks. In IGN, these broad H3K4me3 peaks are centered on the gene body, whereas in the embryo, the sharp peaks are located on promoters (Fig. 5E). This observation suggests that H3K4me3 is actively remodeled at a large fraction of the genes across this developmental transition. We refer to these two patterns as “gene body” and “promoter,” respectively. Because this comparison used data sets from studies in different laboratories using different sources of H3K4me3 antibodies, we confirmed that one antibody could detect both patterns by performing embryonic ChIP-seq with the antibody used in IGN, which reproduced the embryonic “promoter” pattern with high correlation between the two antibodies in the two embryo samples (Supplemental Fig. S7B). Additionally, to confirm that this remodeling is specific to H3K4me3, we compared its profile to H3K27ac, which is also associated with active gene expression and is usually restricted to promoters. Unlike H3K4me3, H3K27ac has a promoter-restricted distribution on approximately the same percentage of genes in each stage (Supplemental Fig. S7C), indicating that dynamic remodeling is not a general characteristic of activating modifications.

In *C. elegans* somatic tissues, broad H3K4me3 domains have been shown to emerge upon aging (Pu et al. 2018), suggesting that the broad pattern in adult IGN might be an indirect consequence of development or aging, and not driven by germline function. We therefore asked whether the broad H3K4me3 domain occurred specifically in IGN, or was found in somatic cells as well, by analyzing H3K4me3 profiles at each developmental stage from whole worms (Jänes et al. 2018), segregated by sets of genes with tissue-specific expression (Serizay et al. 2020). Strikingly, the H3K4me3 pattern shifted from promoter to gene body over development for germline-specific and ubiquitously expressed genes, which are also expressed in the germline, but not for genes expressed specifically in somatic tissues (Supplemental Fig. S7D,E). These observations indicate that the appearance of broad H3K4me3 peaks on gene bodies likely occurs primarily in the germline.

The maintenance of a strict H3K4me3 pattern around the promoter has been recently associated with SET-9 and SET-26, two closely related H3K4me3 readers. Loss of both SET-9 and SET-26 function led to the spreading of H3K4me3 domains around germline-expressed genes, as measured in whole animals (Wang et al. 2018). To investigate whether SET-9 and SET-26 play a role in shaping H3K4me3 specifically during oogenesis, we looked at the overlap between shared gene targets of both SET-9 and SET-26, as well as genes associated with H3K4me3. However, genes marked with H3K4me3 in IGN that were not targeted by the two SET proteins still remodeled their H3K4me3 pattern during the transition from IGN to oocyte to embryo (Supplemental Fig. S7F,G). Thus, SET-9 and SET-26 are not essential for the remodeling of the H3K4me3 pattern from gene body spanning in IGN to promoter-restricted in embryos.

### H3K4me2/3 remodeling is initiated in oogenesis to define genes with early expression in embryos

Based on H3K4me3 remodeling in mouse oocytes (Dahl et al. 2016), we initially speculated that H3K4me3 remodeling occurred upon transcriptional reactivation of the zygotic genome in the early embryo, which is initiated at the four-cell stage and gradually increases over the next several cell divisions before reaching steady-state levels (Robertson and Lin 2015). To more precisely define when H3K4me3 patterns change during the transition between IGN and embryo, we added H3K4me3 data from isolated oocytes and early embryos (Liu et al. 2011; Tabuchi et al. 2018) to investigate intervening developmental time points (Supplemental Fig. S8A–C). Contrary to expectations, H3K4me3 levels at the promoter begin to increase in oocytes well before zygotic genome activation (Supplemental Fig. 6A). Moreover, because oocytes are transcriptionally silent, this result indicates that remodeling does not require ongoing transcriptional activation. Indeed, H3K4me3 levels reach a zenith in early embryos before subsiding later during embryogenesis, once transcription has become fully engaged.

**Figure 6.**
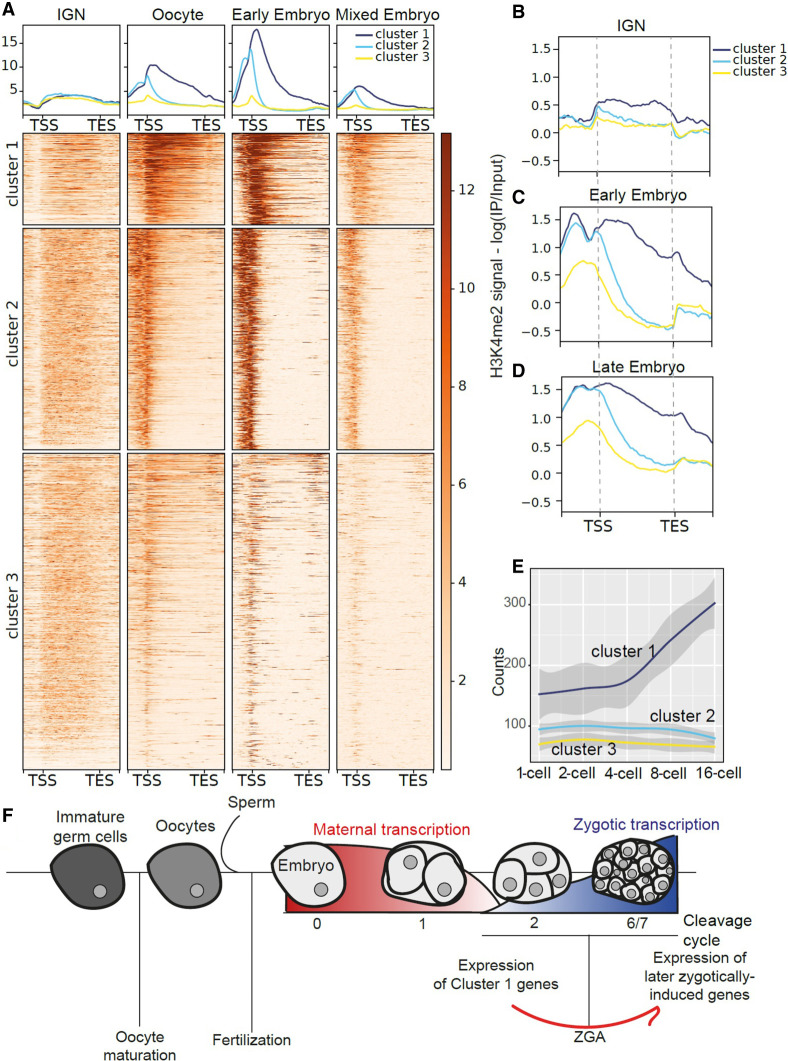
H3K4me3 shows dynamic remodeling during oogenesis. (*A*) Metagene plots and heatmaps of H3K4me3 signal in IGN (Han et al. 2019), oocytes (Tabuchi et al. 2018), early embryos (Tabuchi et al. 2018), and mixed embryos (Beurton et al. 2019). The heatmap represents H3K4me3 signal along genes that are marked in all developmental stages: Genes are ordered based on signal intensity, and each row represents the same gene in all panels. Genes were clustered using the *k*-means clustering method. (*B*–*D*) Metagene plots of H3K4me2 signal in IGN and early embryos (Liu et al. 2011) and late embryos (Samsudin 2017). (*E*) Plot of early embryonic expression (Tintori et al. 2016) for genes belonging to the three clusters. *Z*-score is plotted. (*F*) Model linking H3K4me3 remodeling during the maternal-to-embryo transition to early expression of cluster 1 genes that leads to more efficient expression of additional genes during zygotic genome activation.

This analysis also distinguished three groups of genes with distinct patterns of timing or remodeling (Fig. 6A). Genes in Cluster 1 showed a strong increase of H3K4me3 at the promoter in oocytes and persisted into embryogenesis; notably, this signal extends partially into the gene body. Genes in cluster 2 also showed a strong increase of promoter-associated H3K4me3 in oocytes, but gene body H3K4me3 was lost simultaneously. In contrast, genes in cluster 3 showed minimal accumulation of promoter H3K4me3 in oocytes or embryos and lost even the low levels over the gene body in embryos.

To better understand how H3K4me3 might show these developmentally dynamic patterns, we investigated the precursor modification H3K4me2 using available data in IGN and early and mixed embryos (Liu et al. 2011). Overall, H3K4me2 levels are low relative to H3K4me3 (Fig. 6, cf. A and B–D regarding different scales of the *y*-axis in metagene panels). In IGN, H3K4me2 is detectable across the gene body only for genes in cluster 1 and is preferentially enriched at the promoters of genes in clusters 2 and 3 (Fig. 6B). Thus, cluster 2 and 3 genes only have H3K4me3 across the gene body, whereas cluster 1 genes have both H3K4me2 and H3K4me3 across the gene body. In early and later embryogenesis, H3K4me2 levels increase more for genes in clusters 1 and 2 relative to cluster 3, similar to the pattern of H3K4me3 remodeling. H3K4me2 remains distributed to some degree over the gene body for genes in cluster 1, whereas remodeling at genes in cluster 2 is more rapidly restricted to the promoter, again similar to the patterns of H3K4me3. Thus, these two highly related modifications generally behave coordinately and, together, establish three distinct patterns for subsets of genes across the maternal-to-embryo transition.

We hypothesized that the various H3K4me2/3 patterns showed by genes in each cluster might lead to differential gene regulation during the initiation of zygotic gene activation in early embryogenesis. We therefore used RNA-seq profiles from precisely staged very early embryos (Tintori et al. 2016) and mapped the average expression of genes in each cluster from the one-cell stage to the 16-cell stage (Fig. 6E). Strikingly, cluster 1 gene expression specifically increases beginning at the four-cell stage, whereas clusters 2 and 3 do not. This observation indicates that genes with a persistent H3K4me2/3 pattern across the gene body are primed for earliest possible expression, concomitant with the onset of zygotic genome activation at the four-cell stage. Notably, genes in cluster 1 encode core transcriptional and translational regulatory proteins, including the vast majority of ribosomal proteins (Supplemental Fig. S8D; Supplemental File S2). Clusters 2 and 3 are enriched for genes that function in various embryonic processes and in germ cells, respectively. Together, these observations suggest that the persistent gene body H3K4me2/3 pattern selectively marks genes that are needed for efficient transcription and translation of the gene expression program during zygotic genome activation to ensure the earliest possible expression in embryogenesis (Fig. 6F).

## Discussion

In this report, we have collected and analyzed epigenetic data from diverse genomic assays to characterize the overall chromatin state of adult hermaphrodite germ cells in *C. elegans*. The distribution of chromatin states across the genome captures known and novel features of genome organization in the germline and identifies germline-specific chromatin states. Distinct chromatin states at various sets of germline-expressed genes point to feedback from post-transcriptional regulatory mechanisms, including small RNA pathways, into epigenetic state. In addition, we found that chromatin state dynamics during the oogenesis-to-embryogenesis transition revealed that most histone modifications remain fairly stable, with the exception of H3K4me3. We have made these chromatin states available for further computational analysis and for viewing via genome browser (Supplemental File S1). This analysis sets the stage for further investigation into the underlying mechanisms that establish these states, as well as the phenotypic consequences to germline development and embryogenesis when these states are disrupted.

Several unique features of germline gene regulation were apparent in this analysis, and certain differences in chromatin states between the germline and soma suggest distinct regulatory mechanisms. For instance, the “strongly inactive TSS” state 5 found only in IGN is characterized by conflicting signals of open chromatin that are marked at the promoter by the silencing modification H3K27me3 (Figs. 1, 2). To a much lesser extent, state 7, primed TSS, also has a similar profile. Genes associated with these states tend to function in signal transduction, neuronal activity, and development (Supplemental Fig. S1D) and mostly do not show enriched expression in the germline. Thus, they might functionally approximate the “poised” chromatin state found in germ cells of other organisms, in which genes expressed in the embryo are placed in a chromatin state in the germline that prevents immediate transcription but facilitates rapid expression postfertilization (Macrae et al. 2023). However, the chromatin hallmarks of poising in mammals are the colocalization of the opposing marks H3K27me3 and H3K4me3 (Macrae et al. 2023), a configuration not found in any germline chromatin state in *C. elegans*. Thus, this observation suggests the existence of an alternate mechanism that uses only H3K27me3 to temporally delay gene expression across the maternal-to-embryo transition.

Additionally, certain chromatin states showed complex correlations between sites of open chromatin, accumulation of multiple active histone marks, and transcript abundance. A priori, one would expect that transcript abundance would be roughly proportional to the level of active histone marks and chromatin accessibility, as seen overall for the germline chromatin states (Fig. 1A,B). However, we found multiple chromatin states with higher or lower transcript abundance than might be expected based on the combined levels of active/inactive modifications (e.g., state 1, pregamete; state 9, spermatogenesis; state 11, oogenesis) (Fig. 3). These instances suggest the existence of “escaper” loci that can be transcribed from inhospitable chromatin environments and/or from extensive post-transcriptional regulation that stabilizes or degrades transcripts. Indeed, many mechanisms of post-transcriptional regulation in the germline have been shown to affect the mitosis-to-meiosis transition, the sperm-to-oocyte switch, and other critical aspects of germline development (Seydoux and Schedl 2001; Frolows and Ashe 2021).

In particular, post-transcriptional regulation through small RNA pathways not only affects target mRNA transcripts but can lead to alterations of chromatin modifications at the genomic locus of those targets. The best-studied example is the AGO protein HRDE-1, which localizes to the nucleus in germ cells and promotes deposition of H3K9me3 at target loci (Seroussi et al. 2023). Other AGO proteins might also have a similar effect, either directly or indirectly, although this possibility has not been thoroughly investigated. H3K9me3 is enriched at HRDE-1 target genes, as expected, but is actually much stronger for the targets of PPW-2, which is in the same WAGO-1 group as HRDE-1 but does not show detectable nuclear localization (Fig. 4). Additionally, AGOs in the ALG-3/4 group, which promote spermatogenesis (Seroussi et al. 2023), display a striking increase of H3K4me3 at target genes specifically in sperm, suggesting a downstream effect of ALG-3/4 on the chromatin state of its targets. Finally, targets of the CSR-1 group show high levels of the active marks H3K36me3 and H3K4me3 in IGN, as expected, because these AGOs target germline-expressed genes. However, for all these genes, H3K36me3 is primarily present only in the germline and begins to decrease in gametes and in embryos, whereas H3K4me3 persists throughout the maternal-to-embryo transition and into later somatic development (Fig. 6A). This observation raises the possibility that CSR-1 is functionally linked to H3K36me3 at its targets in the germline. CSR-1, which is perinuclear throughout most of germline differentiation, enters the nucleus in oocytes and early embryos (Claycomb et al. 2009), when H3K36me3 levels are declining. CSR-1 might therefore play a direct role in altering this modification during this time. The mechanisms by which most AGO proteins affect these marks directly or indirectly is currently unclear and will require functional investigation of many of the different histone methyltransferases and demethylases expressed in the germline.

One of the most striking observations of this analysis is the extensive remodeling of H3K4me2 and H3K4me3 during the maternal-to-embryo transition (Figs. 5, 6). This remodeling is twofold: The set of genes enriched for H3K4me2/3 changes, and the specific distribution pattern of H3K4me2/3 across genes changes from a broad “gene body” pattern to a focused accumulation at promoters. H3K4me3 has been primarily described as a promoter mark, but recent studies have found instances in which it covers larger genomic regions, including during aging in *C. elegans* (Dahl et al. 2016; Pu et al. 2018). H3K4me3 is also remodeled from broad peaks to promoter peaks during the mouse maternal-to-zygotic transition (Dahl et al. 2016) but appears to be dependent on transcriptional reactivation of the embryonic genome, whereas in *C. elegans*, this remodeling occurs during a developmental window in which there is minimal to no active transcription. Intriguingly, a recent report showed that in *C. elegans*, the canonical histone H3 is replaced with the variant H3.3 at the approximate time of this H3K4me3 remodeling, during oogenesis (Gleason et al. 2023). H3.3 is the dominant form specifically during early embryogenesis, until it is again replaced by canonical H3 later in development. This time frame corresponds with the H3K4me3 remodeling that we report here, suggesting a possible connection; however, we did not see a significant overlap in the genes reported to have this H3 remodeling event and those we found to have H3K4me2/3 remodeling. Additionally, two H3K4me3 readers, SET-9 and SET-26, have been shown to restrict the breadth of H3K4me3 peaks in the *C. elegans* germline and to preserve fertility (Wang et al. 2018). However, genes associated with H3K4me3 that are not targeted by SET-9 and SET-26 still showed remodeling of the histone mark (Supplemental Fig. S7F,G), showing that these two histone readers are not the essential players in this process. The fact that H3K4me2 shows similar remodeling to H3K4me3 suggests the involvement of a histone methyltransferase(s) capable of adding both me2 and me3 to H3K4, such as SET-2 or SET-16 (Xiao et al. 2011). Overall, however, the mechanisms that drive specificity and timing of changes to H3K4me3 distribution during the IGN and oogenesis are currently unknown but will be critical to understand the functional consequences of this remodeling.

In the maternal germline, transcription by RNA polymerase II is repressed sometime during oogenesis and does not resume until the onset of zygotic genome activation in early embryogenesis, between the four- and 28-cell stage in *C. elegans* (Robertson and Lin 2015). A priori, the most likely expectation is that H3K4me2/3 remodeling would be coupled to the renewed onset of transcriptional activation in embryos, but instead, we found that it occurs before or concomitant with the transcriptional shutdown during oogenesis in the maternal germline. Moreover, the subset of remodeled genes with a persistent gene body H3K4me2/3 pattern are enriched for functions in core gene regulatory processes required in all cells (cluster 1), whereas genes with germline-specific functions (e.g., meiosis) that are not expressed in the embryo (cluster 3) do not show this remodeling. This observation suggests that the remodeling “pre-establishes” expression in the very early embryo specifically for selected essential genes that, in turn, promote the expression of subsequent embryonic genes during the onset of zygotic gene expression. The mechanisms that select this set of genes for remodeling are unknown. The distinct H3K4me3 patterns in IGN and embryos also suggest the possibility that transcriptional regulation by RNA polymerase II might be very different before and after the maternal-to-embryo transition.

Together, this integrated analysis of chromatin state in the *C. elegans* germline establishes an initial overview that provides insight into gene regulatory processes and germline biology. However, this approach is limited in some aspects. The catalog of histone modifications is far from complete, and the included genomic assays are restricted to linear chromatin so that any correlations between the chromatin state and three-dimensional organization in the germline are unknown. Although the use of IGN greatly improves specificity and resolution, it represents an aggregate picture of nuclei at multiple points along the early germ cell differentiation trajectory before gametogenesis. In the future, technologies that simultaneously measure chromatin accessibility and RNA levels in single cells will refine the dynamics of chromatin state during germ cell differentiation. Regardless, characterizing adult IGN chromatin states using multiple histone modifications and chromatin accessibility, as we have performed here, provides a powerful foundation on which to pursue finer-detailed characterizations and more mechanistic studies.

## Methods

### Data sets and genome versions

Throughout this study, we used the wBcel235/ce11 (WS235) version of the *C. elegans* genome, as well as WormBase WS286 genome annotations, with coordinates backlifted to WBcel235/ce11 (WS235).

Data sets used in these analyses were all extracted from published work and can be accessed from the NCBI Gene Expression Omnibus (GEO; https://www.ncbi.nlm.nih.gov/geo/) at the following accession numbers.

- H3K4me3 and H3K27ac ChIP-seq data sets from IGN from wild-type (strain VC2010) and *glp-1(q224)* mutants at the young adult stage used in this analysis refer to accession number GSE117061 (Han et al. 2019), whereas H3K36me3 and H3K27me3 ChIP-seq data sets refer to GSE147401 (McManus et al. 2021).
- H3K9me3 data from IGN samples refer to Sanchez et al. (2023), whereas data from *glp-1* young adult mutants refer to GSE141347 (Schwartz-Orbach et al. 2020).
- H3K4me2 data from germline nuclei refer to GSE50290 (Liu et al. 2011).
- ChIP-seq data sets from mixed embryos refer to GSE114440 (Jänes et al. 2018), GSM3148388 (Beurton et al. 2019), and GSE168923 (Delaney et al. 2022); ChIP-seq data sets from early embryos, sperm, and oocytes were all obtained from GSE115709 (Tabuchi et al. 2018); H3K4me2 data from early embryos refer to GSE49733 (Liu et al. 2011); and data from late embryos refer to the published dissertation of Samsudin (2017) (GSE94639).
- ATAC-seq data from IGN were taken from GSE232139 (Sanchez et al. 2023), whereas ATAC-seq data from mixed embryos and *glp-1* mutants at young adult stage were taken from GSE114440 (Jänes et al. 2018).
- RNA-seq data from IGN and *glp-1* mutants acquired at the young adult stage were obtained from GSE117061 (Han et al. 2019).
- Developmental *C. elegans* RNA-seq data were obtained from Boeck et al. (2016).
- Single-cell RNA-seq data from early embryos were obtained from Tintori et al. (2016).

### ChIP-seq experiments

Embryonic stages were collected from VC2010 worms after bleaching and arresting in M9 at 20°C until the desired stage was visualized. Worm synchronization was achieved by bleaching and L1 starvation. Arrested L1s were plated on peptone-enriched NGM plates seeded with OP50 bacteria and grown at 20°C. Adult worms were then bleached for early embryo collection. ChIP was conducted as previously described (Kudron et al. 2018). Briefly, worm samples were cross-linked with 2% formaldehyde for 30 min at room temperature and then quenched with 1 M Tris (pH 7.5). The pelleted worms were subsequently flash-frozen in liquid nitrogen and stored at −80°C. Samples were sonicated using a microtip to achieve mostly 200- to 800-bp DNA fragments. Finally, 5 μg of anti-H3K4me3 (Active Motif 61379) was incubated with each sample overnight at 4°C with rotation.

### ChIP-seq and ATAC-seq data processing

Because the data used in this study came from many different sources, we reprocessed all data identically as follows. The raw data in FASTQ format were aligned to the reference genome ce11 using Bowtie 2 (Langmead and Salzberg 2012) and then converted to SAM, sorted, and filtered for uniquely aligned reads (-q 10) using SAMtools (Li et al. 2009). Peaks were called with MACS2 (Feng et al. 2012) using the standard options (-q 0.001 –nomodel –extsize 150). bigWig files were obtained using the bigwigCompare function in the deepTools software (Ramírez et al. 2014) and read-normalized on input files.

Metagene plots were obtained using the computeMatrix and plotProfile tools in deepTools using the reference-point mode (‐‐regionBodyLength 3000 ‐‐beforeRegionStartLength 2000 ‐‐afterRegionStartLength 2000 ‐‐binSize 50).

Read counting was performed using the dbacount function in the DiffBind package (Wu et al. 2015); Annotation of genomic regions and GO enrichment were performed using the ChIPseeker and ClusterProfiler packages (Yu et al. 2012, 2015).

To visualize the distribution of histone marks along the chromosomes, we used the rtracklayer package for importing all bigWig files (Lawrence and Gentleman 2009).

### Annotation of chromatin states

Annotation of chromatin states was performed on histone marks (ChIP-seq) and chromatin accessibility (ATAC-seq) data using the ChromHMM software (Beltran et al. 2019). The program partitions the genome in 200-bp intervals while aligning it to the reference genome of interest, assesses the presence or absence of a specific mark on each bin, uses a HMM on the resulting calls to learn a chromatin-state model, and, finally, obtains the annotation of each state occurrences on the genome. The standard output contains (1) a mark emission table containing the probability of each mark in a specific state, (2) a heatmap containing the percentage of distribution for each chromatin state around defined genomic regions, (3) the emission signal of each chromatin state around the TSS, and (4) the emission state of each chromatin state around the transcription end site (TES).

States were named on the basis of the combination of marks visualized, the probability of each state at annotated genomic regions, and the distribution relative to the TSSs and TESs (Ernst and Kellis 2012).

The analysis was performed on both germline and somatic data using the “independent” mode. We tested multiple numbers of states and selected a 12-state model for all further analyses, which best represented the diverse relationships between patterns of histone marks, open chromatin, and genomic features.

### Transcript integrity

RNA degradation was measured by a calculation of “transcript integrity,” which determines the frequency of defined ends of transcripts compared with shortened or incomplete transcripts, using RseQC software for Linux (Wang et al. 2016).

### Data visualization

To visualize our results the following R (R Core Team 2021) and Python packages were used:

- Gviz and KaryotypeR packages for the chromosomal views of chromatin states (Hahne and Ivanek 2016; Gel and Serra 2017);
- the Venneuler package for Venn diagrams;
- the deepTools package for epigenetic profile plots around specific genes (both metagene plots and heatmaps), with the computeMatrix, plotProfile and plotHeatmap functions (Ramírez et al. 2014);
- the ComplexHeatmap package for heatmaps showing the average signal of epigenetic marks around each chromatin state, as well as embryo–IGN correlations (Gu et al. 2016); and
- the ReactomePA package for plotting GO results (Yu and He 2016).

### Gene lists

Genes with enriched expression in sperm, oocytes, and pregametic cells (Fig. 2) were taken from Lee et al. (2017), whereas sperm genes targeted by the piRNA pathway were obtained from Cornes et al. (2022). Genes with tissue-specific expression (Fig. 4) were acquired from Serizay et al. (2020). The lists of small RNA pathway target genes were taken from Seroussi et al. (2023). Finally, the list of SET-9 and SET-26 target genes was taken from Wang et al. (2018).

## Data access

All raw and processed sequencing data generated in this study have been submitted to the NCBI Gene Expression Omnibus (GEO; https://www.ncbi.nlm.nih.gov/geo/) under accession number GSE236076.

## Supplementary Material

Supplement 1

Supplement 2

Supplement 3
