# Supplementary material for: Characterization of the distribution and dynamics of chromatin states in the C. elegans germline reveals substantial H3K4me3 remodeling during oogenesis
Source: Genome Res. 2024 Jan;34(1):57–69. doi: 10.1101/gr.278247.123 (PMC10903938; doi:10.1101/gr.278247.123)
Supplement: Supplement 1 [file Supplemental_Figures.pdf]

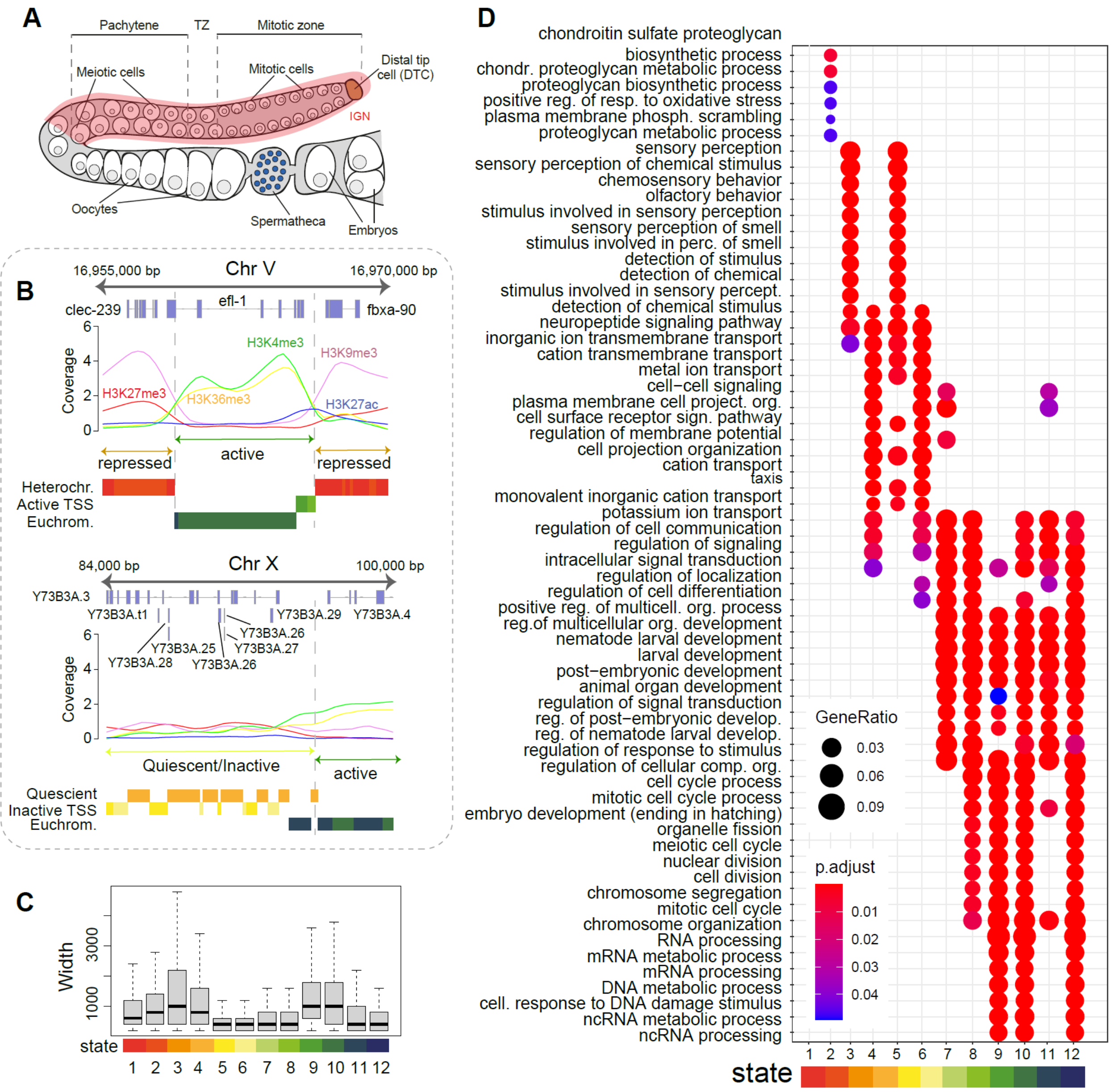

Supplemental Figure 1. Characteristics of germline chromatin state A) Representation of the *C. elegans* germ line. The region from which nuclei are isolated for IGN (Han et al., 2019) are highlighted in red. B) Genomic visualization of the chromatin states along a portion of chromosome V (above) and chromosome X (below). C) Width in base pairs of the genomic sections for each state. D) Enriched categories for genes contained in each chromatin state using Gene Ontology analysis; only significantly enriched categories (with adjusted p-value < 0.1) were plotted as a dotplot. Both gene ratio and adjusted p-values are included as shape size, and color code respectively.

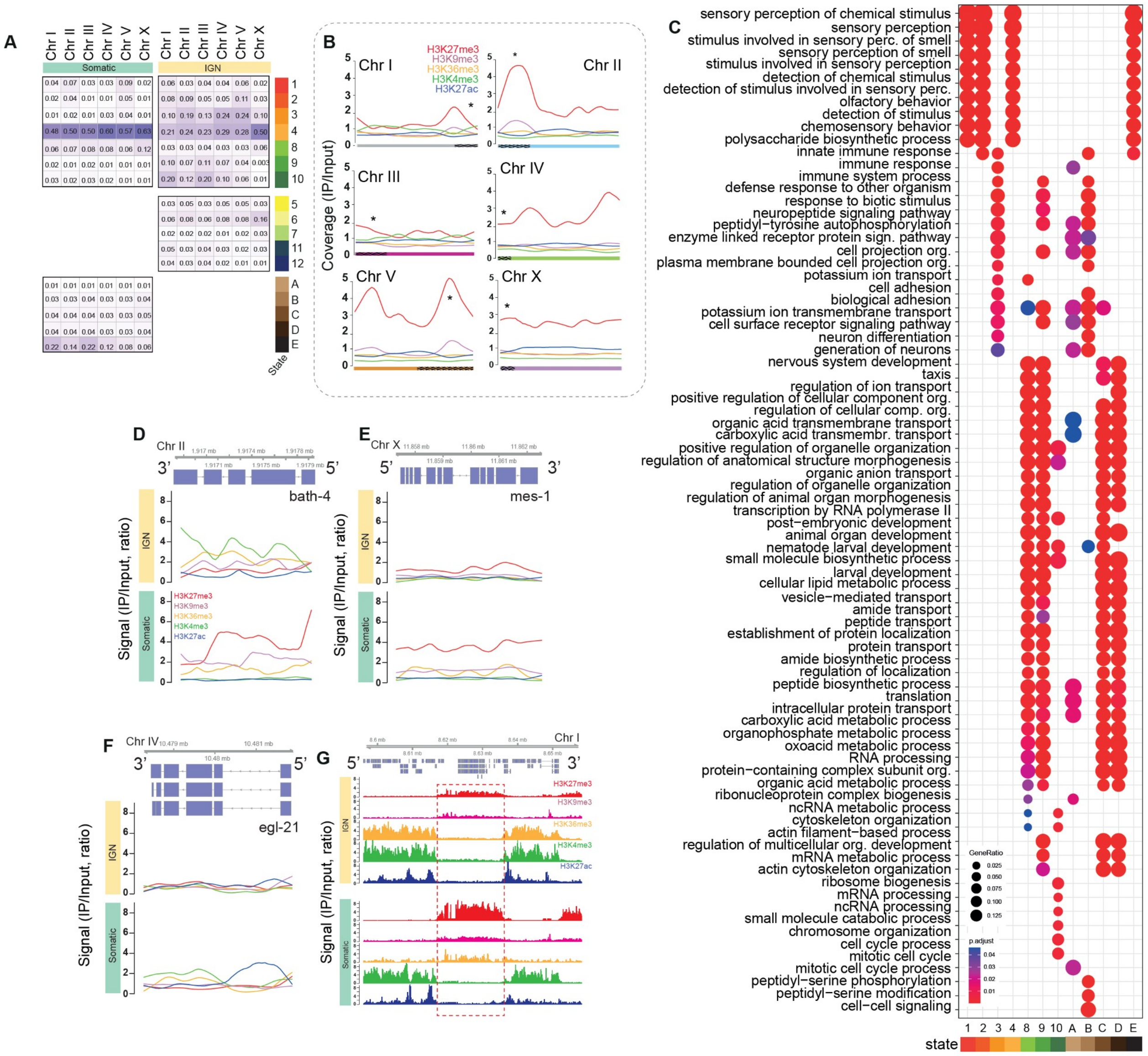

Supplemental Figure 2. **Chromatin states unique to germ line and soma.** A) Percentage of the individual chromosomes in each chromatin state for germline and somatic tissues. Chromatin states are divided in “shared” states (above), “only-IGN” states (center), and “only-soma” states (below). B) Distribution of normalized signal (as IP/input ratio) for each epigenetic mark along each chromosome in the soma. C) Enriched GO categories for genes contained in each somatic chromatin state; only significantly enriched categories (with adjusted p-value < 0.1) were plotted. Gene ratio and adjusted p-values are included as shape size, and color code respectively. D-F) Examples of genes with altered epigenetic profile between germline and soma: *bath-4* (D), *mes-1* (E), and *egl-21* (F). G) Genome visualization of the chromatin states along a portion of chromosome I shows additional epigenetic differences between germ line and soma.

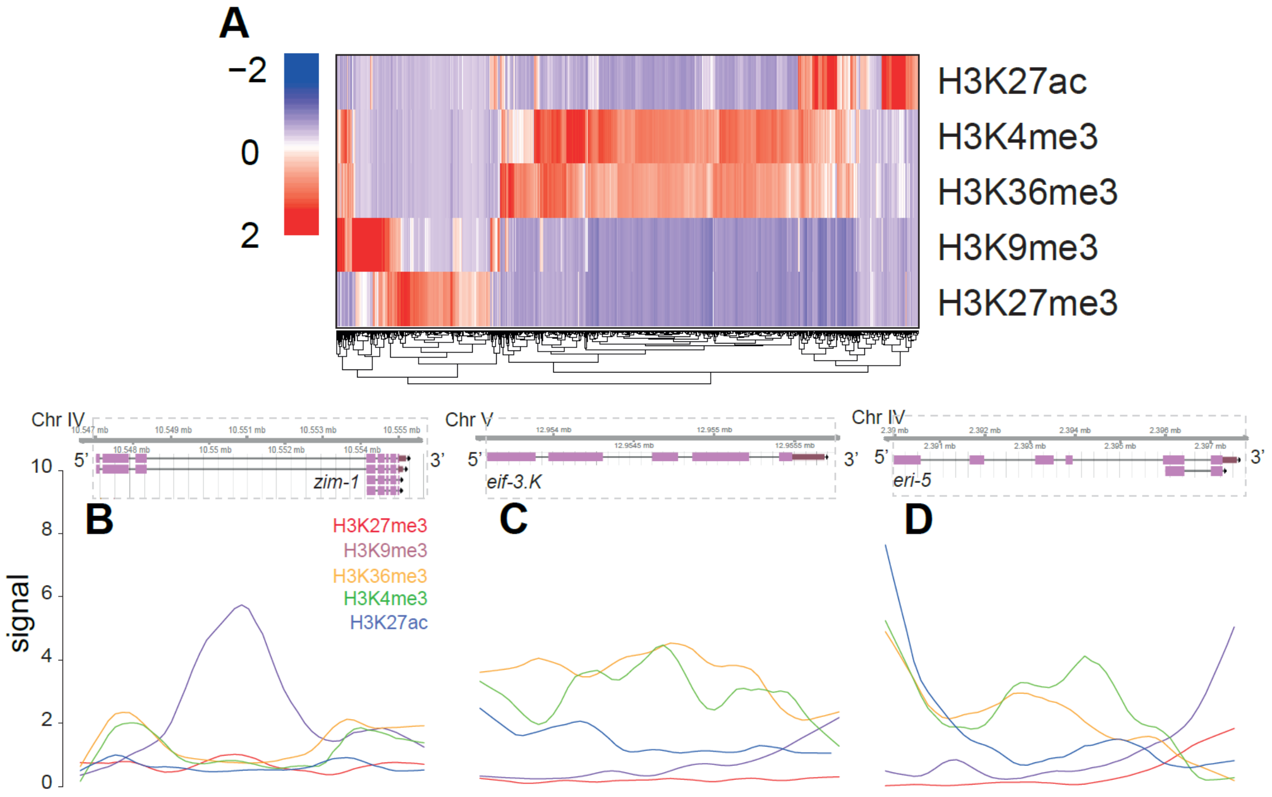

Supplemental Figure 3. **Chromatin states of classes of germline-expressed genes in IGN - pregamete.** A) Heatmap of individual histone modification for genes with pregamete-enriched expression. Values are centered on the mean and scaled on the standard deviation. B-D) Genome visualization of histone mark levels across representative loci *zim-1* (B), *eif-3.K* (C), and *eri-5* (D).

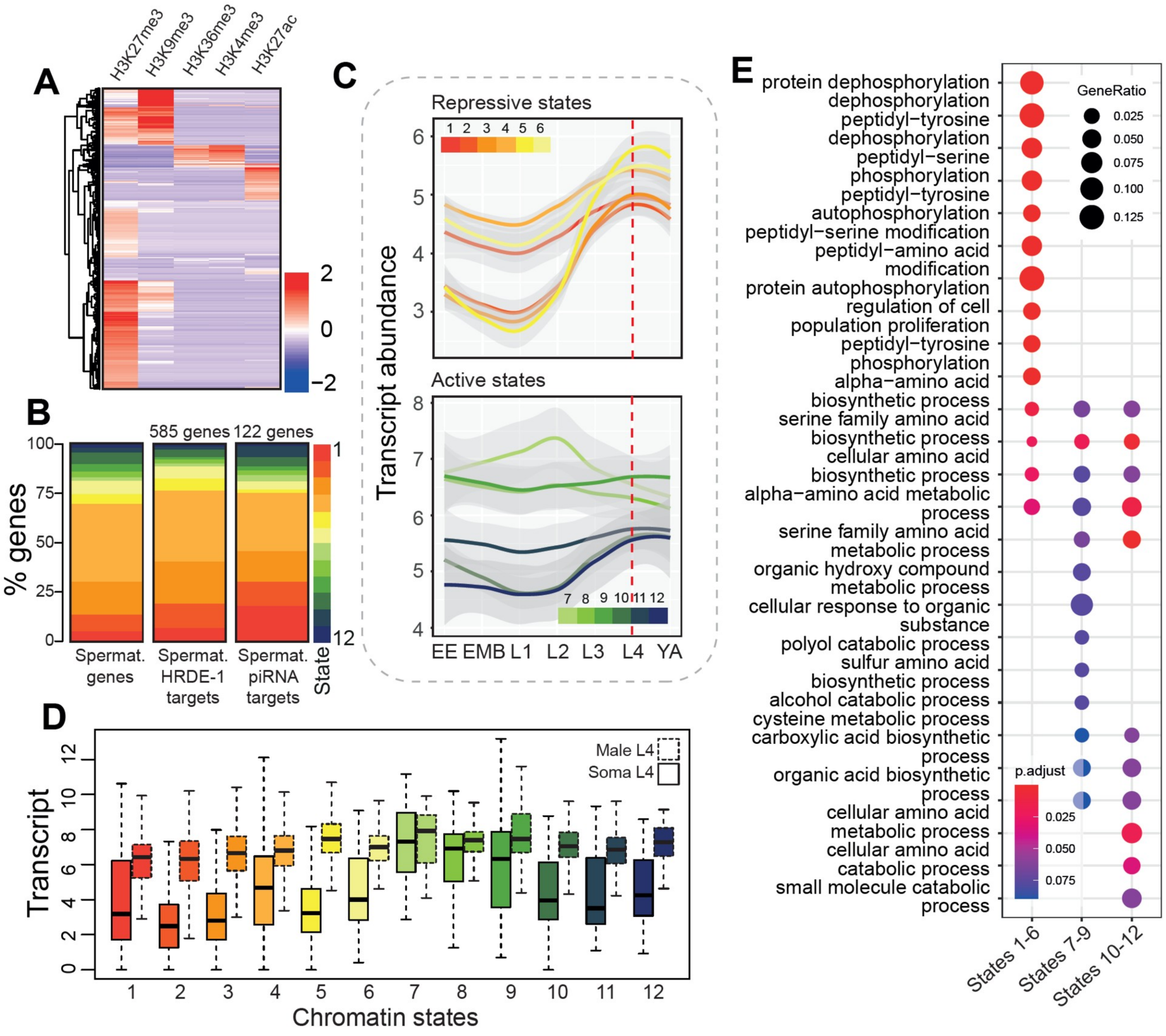

Supplemental Figure 4. **Chromatin states of classes of germline-expressed genes in IGN - spermatogenesis.** A) Heatmap of individual histone modifications for genes with spermatogenesis-enriched expression. Values are centered on the mean and scaled on the standard deviation. B) Distribution of chromatin states for all genes with spermatogenesis-enriched expression (left), only those that are targets of HRDE-1 (middle), and only those that are piRNA targets (right) (Cornes et al. 2022). C) Gene expression developmental profiles for genes with spermatogenesis-enriched expression using transcriptomic data (Boeck et al. 2016), with inactive states 1-6 separated from active states 7-12. Log normalized counts are visualized. D) Gene expression profiles for genes with spermatogenesis-enriched expression in each state in somatic tissues from hermaphrodites (black outline) and males (dotted outline) at the L4 stage. E) Gene Ontology analysis of genes with spermatogenesis-enriched expression for combined chromatin states - inactive (states 1-6), expressed (states 7-9), and active but not expressed (states 10-12). Both adjusted p-value and gene ratio are included.

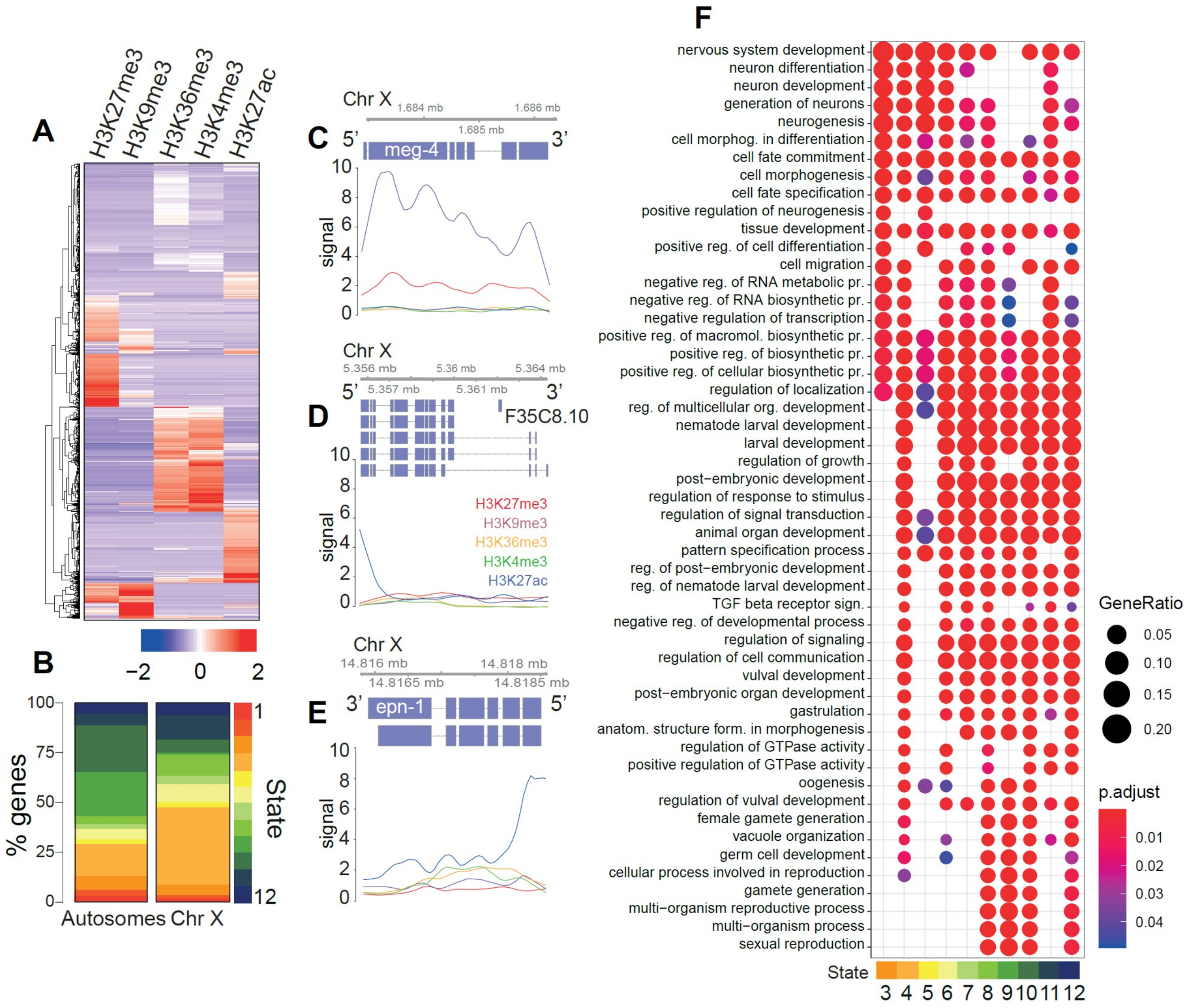

Supplemental Figure 5. **Chromatin states of classes of germline-expressed genes in IGN - oogenesis.** A) Heatmap of individual histone modification for genes with oogenesis-enriched expression. Values are centered on the mean and scaled on the standard deviation. B) Chromatin states for oogenesis-enriched genes on autosomes and chromosome X. C-E) Genomic visualization of histone mark levels along *meg-4* (C), *chtl-1* (D), and *epn-1* (E). The genomic portion of the chromosome, the annotated genes, as well as the related chromatin state, are visualized. F) Gene Ontology analysis of genes with oogenesis-enriched expression for chromatin states 3-12. Adjusted p-value and gene ratio are included.

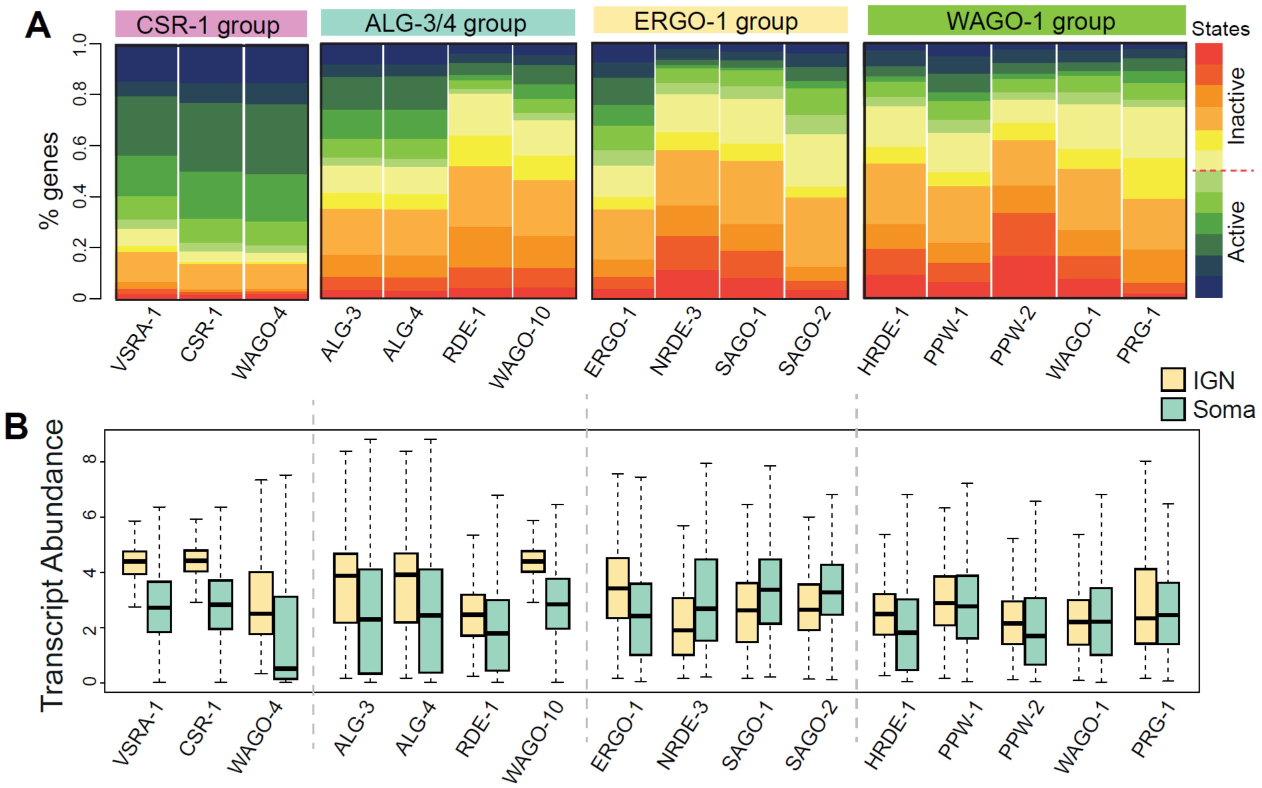

Supplemental Figure 6. Chromatin states of AGO target genes. A) Distribution of chromatin states along targets of AGO proteins in all AGO groups (Seroussi et al. 2023). B) Transcript abundance (FPKM) of these targets in IGN and soma.

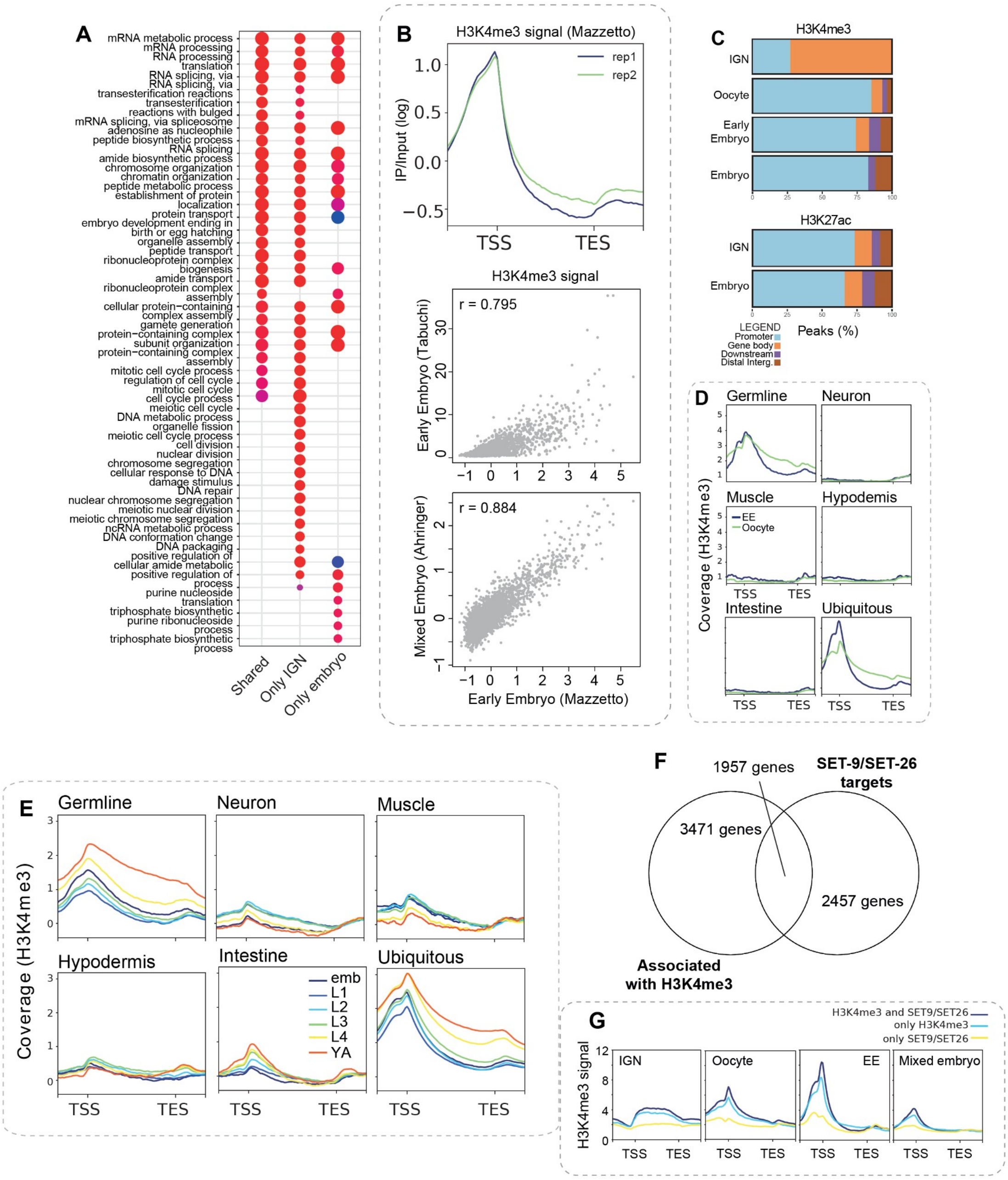

Supplemental Figure 7. **H3K4me3 profiles in IGN and embryo.** A) GO analysis of genes marked by H3K4me3 in both IGN and embryos ("shared"), genes marked only in IGN ("Only IGN"), and genes marked only in embryos ("Only embryo"). Adjusted p-value and gene ratio are included. B) Correlation plot of our antibody embryo data compared to published embryo data. From top to bottom: metagene plots of early embryo ChIP-seq data obtained independently in this study using anti-H3K4me3 antibody (61379, Active Motif) (top panel); correlation plot between H3K4me3 signals; the x-axis represents the early embryo data obtained in this study, and the y-axis represents Tabuchi et al. (2018) early embryo data (middle panel); correlation plot between H3K4me3 signals; the x-axis represents the early embryo obtained in this study, and the y-axis represents the Beurton et al. (2019) mixed embryo data (bottom panel). C) Distribution of H3K4me3 and H3K27ac across gene features at multiple developmental stages. D) Metagene plots

of H3K4me3 signal in early embryos and oocytes for genes with tissue-specific expression (Serizay et al. 2020). E) Metagene plots of H3K4me3 signal during larval development for genes with tissue-specific expression. Normalized IP/input ratio is plotted for upstream region (TSS-1000bp), gene body, and downstream regions (TES+1000 bp). F-G) Comparison between H3K4me3 remodeling during oogenesis and SET-9/SET-26 targets. F) Overlap between genes associated with H3K4me3 in IGN, and SET-9/SET-26 targets as defined by Wang et al. (2018). G) Metagene plots of H3K4me3 signal around genes associated with H3K4me3 and targeted by SET-9/SET-26 (blue), genes only associated with H3K4me3 (cyan), and genes only targeted by SET-9/SET-26 (yellow) for IGN, oocyte, early embryo, and mixed embryo datasets. Normalized signals (as IP/input ratio) were used for plotting over upstream region (TSS-1000bp), gene body, and downstream regions (TES+1000 bp).

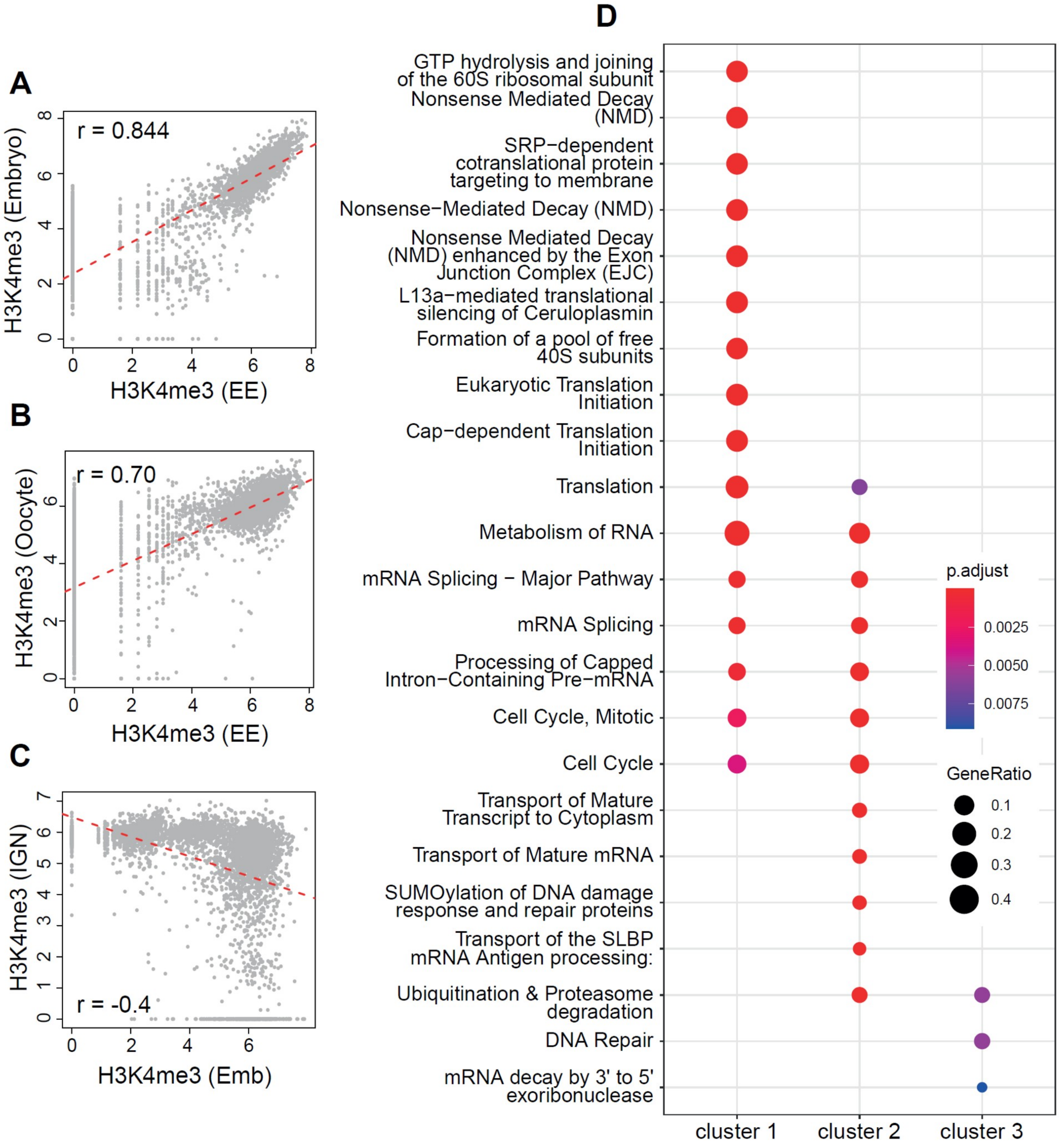

Supplemental Figure 8. H3K4me3 exhibits dynamic remodeling during oogenesis A-C) Correlation plots of H3K4me3 signal (as normalized IP/input log ratio) between different stages of development, in 500bp bins. Pearson correlation and slope are also shown. A. Embryo (X-axis) vs oocyte (Y-axis), B Embryo vs early embryo, C. Embryo vs IGN. Embryo data from (Beurton et al. 2019), oocyte and early embryo from (Tabuchi et al. 2018), and IGN from (Han et al. 2019). D) GO analysis of genes marked by H3K4me3 for each Cluster 1, 2, and 3. Adjusted p-value and gene ratio are included.
