# Supplementary material for: Characterization of the distribution and dynamics of chromatin states in the C. elegans germline reveals substantial H3K4me3 remodeling during oogenesis
Source: Genome Res. 2024 Jan;34(1):57–69. doi: 10.1101/gr.278247.123 (PMC10903938; doi:10.1101/gr.278247.123)
Supplement: Supplement 2 [file Supplemental_File_1.zip › Supplemental_File_1/Supplemental_File_1/README.rtf]

Supplemental_File_1. Spreadsheet containing chromatin state coordinates and identification for browser viewing and for analysis. 
